# Deep Molecular and In Silico Protein Analysis of p53 Alteration in Myelodysplastic Neoplasia and Acute Myeloid Leukemia

**DOI:** 10.3390/cells11213475

**Published:** 2022-11-02

**Authors:** Kristóf Madarász, János András Mótyán, Judit Bedekovics, Zsófia Miltényi, Anikó Ujfalusi, Gábor Méhes, Attila Mokánszki

**Affiliations:** 1 Department of Pathology, Faculty of Medicine, University of Debrecen, 4032 Debrecen, Hungary; 2 Department of Biochemistry and Molecular Biology, Faculty of Medicine, University of Debrecen, 4032 Debrecen, Hungary; 3 Department of Hematology, Faculty of Medicine, University of Debrecen, 4032 Debrecen, Hungary; 4 Department of Laboratory Medicine, Faculty of Medicine, University of Debrecen, 4032 Debrecen, Hungary

**Keywords:** myelodysplastic neoplasias (MDS), acute myeloid leukemia (AML), *TP53* gene, p53 protein, next-generation sequencing (NGS), in silico bioinformatic analysis

## Abstract

Background: Mutation of the *TP53* gene is one of the major drivers of myelodysplastic neoplasias (MDS) and acute myeloid leukemia with myelodysplasia-related changes (AML-MR). *TP53* mutations present in these hematopoietic malignancies form a distinct molecular genetic cluster with a worse prognosis than without the alteration. However, besides well-characterized hot-spot variants, a significant proportion of *TP53* alterations are of uncertain clinical significance. Methods: To enlighten so far unknown aspects, bone-marrow samples from altogether 77 patients are analyzed retrospectively with the diagnosis of AML-MR (26 cases), MDS-IB (12 cases), and MDS-LB (39 cases) according to WHO 2022 guidelines. Next-generation sequencing results are correlated with histological, cytogenetic, and survival data. Results: Twenty out of the 30 *TP53* mutation types detected by NGS are not categorized in current public databases; thus, their clinical significance remained mysterious. Because of the interpretation difficulties and the absence of clinical correlations, pathogenicity is established based on in silico approaches. The 12 pathogenicity classification systems, as well as protein stability, protein–DNA, protein–protein interaction, and post-translational modification analyses are applied. We found statistically significant differences between AML/MDS groups considering p53 pathogenicity, protein structural changes, and overall survival. The largest number of abnormalities with the most severe consequences are found in AML-MR cases. Conclusions: These molecular and in silico protein data further support that MDS with increased-blast (MDS-IB) is an intermediate group between AML-MR and MDS with low-blast (MDS-LB) patients, which frequently progresses to AML and is therefore considered a pre-leukemic condition.

## 1. Introduction

Molecular genetic characterization of clonal hematopoiesis of indeterminate potential (CHIP), myelodysplastic neoplasias (MDS), and acute myeloid leukemia (AML) using next-generation sequencing (NGS) has significantly improved our understanding of the pathogenetic aberrations in the background of these malignancies [1]. According to WHO statistics, the incidence of MDS is 3–5 per 100,000 people, rising to 20 over 70 years old and 25–35% of these cases are transformed into AML. MDS and AML with myelodysplasia-related changes (AML-MR) with *TP53* mutations represents a distinct molecular cohort with a uniformly poor prognosis. While data for many specific changes accumulate regarding CHIP/MDS and MDS/AML transition, the clinical role of *TP53* mutations seems to be already well established. A greater number of mutations with higher allele frequencies are rather supportive of the diagnosis of MDS.

The *TP53* gene is one of the most frequently mutated genes in cancer-related diseases [2,3], being found in more than 50% of human tumors [4]. The functions of the p53 protein in inhibiting harmful cell division, such as regulating the cell cycle, apoptosis, and DNA repair, are vital for the normal functioning of the cell, hence its name as the “Gatekeeper” of the cell cycle [5]. For this reason, modification of the p53 protein is one of the drivers of cancer development [6]. Many DNA damage events can activate the p53 protein, such as hypoxia, heat shock, or other stress signals. The activation of p53 occurs through modifications in the N- and C-terminal domains of the protein, which are mainly serine and threonine phosphorylations in the N-terminal transactivator domain (TAD) [7].

In addition, *TP53* mutations are independent covariates for inferior overall survival (OS) in MDS with the available disease-modifying therapies (such as treatment with hypomethylating agents and allogeneic hematopoietic stem cell transplantation.) Importantly, the mechanisms by which *TP53* mutations drive these inferior outcomes have not been resolved. Several *TP53*-dependent and independent clones may coexist, highlighting the broad genetic intratumoral heterogeneity of human tumors [8]. Sensitive sequencing techniques should be used in MDS because even small subclones present at the initial diagnosis can later expand to therapeutic resistance.

OS of patients with *TP53* mutation was shorter than patients with wild-type *TP53* status (9 versus 66 months), and *TP53* was found to be the strongest predictor for OS [8]. No other gene mutation was significantly associated with OS in univariate analyses [9]. The prognostic significance of *TP53* mutations depends in part on their variant allele frequency (VAF), with smaller clones having a less adverse impact, and patients with a lower VAF having better survival [10,11]. Consideration of multiple clinical, cytogenetic, and molecular features identifies *TP53* mutation as the most significant prognostic factor in patients with MDS, yet it remains the only marker not routinely assessed in clinical practice. *TP53* mutational burden evaluation can therefore predict prognosis and identify the subgroup of patients eligible for targeted therapy.

The aims of the study were (i) to investigate the clonal heterogeneity of MDS and AML bone marrow samples based on *TP53* gene mutation status, (ii) to find a correlation between mutation status and the severity of hematopoietic disorders, and (iii) to investigate the alterations of p53 protein upon mutations in silico. For this purpose, after histologic examination, NGS analysis targeting the *TP53* gene (Accel-Amplicon Comprehensive *TP53* panel) was performed on all samples of 77 AML-MR, MDS with increased-blast (MDS-IB), and MDS with low-blast (MDS-LB) patients. Because of the interpretation difficulties of detected aberrations, different pathogenicity scoring systems were compared. In addition, several in silico sequence- and structure-based analyses were performed to investigate the stability changes of p53 at the protein level. We examined the protein–protein and protein–DNA interactions, as well, due to the versatile function of the p53 protein.

## 2. Materials and Methods

### 2.1. Patients and Samples

Patients were managed and treated at the Department of Hematology at the University of Debrecen. Formaldehyde-fixed paraffin-embedded bone-marrow biopsy tissue (FFPE) samples were analyzed retrospectively from altogether 77 patients reclassified with AML-MR (26 cases), MDS-IB (12 cases), and MDS-LB (39 cases) according to WHO 2022 guidelines at the Department of Pathology at University of Debrecen. Hematoxylin and eosin (H & E) stained slides were analyzed by pathology specialists. Cytogenetic analysis was performed as the routine diagnostic procedure. All protocols have been approved by the author’s respective Institutional Review Board for human subjects (IRB reference number: 60355-2/2016/EKU and IV/8465-3/2021/EKU). Sampling was agreed upon and supported by written consent. This study was managed according to the Declaration of Helsinki.

### 2.2. Immunohistochemistry

After the H & E examination, p53 (clone Do-07 Dako, Agilent Technologies Company, Santa Clara, CA, USA) immunohistochemical analysis (IHC) was also performed. IHC positivity was defined when p53 staining intensity was high (3+) with at least 10% of positive cells.

### 2.3. DNA Isolation

QIAamp DNA FFPE Tissue Kit (Qiagen, Hilden, Germany) was applied for FFPE tissues genomic DNA (gDNA) extraction. The isolations were carried out according to the manufacturer’s instructions and the gDNA was eluted in 50 µL elution buffer. The DNA concentrations were measured using the Qubit dsDNA HS Assay Kit in a Qubit 4.0 Fluorometer (Thermo Fisher Scientific, Waltham, MA, USA).

### 2.4. Next-Generation Sequencing

After the fragmentation of the genomic DNA, libraries were created by the Accel-Amplicon Comprehensive *TP53* panel (Swift Biosciences, Ann Arbor, MI, USA). The MiSeq System (MiSeq Reagent kit v3 600 cycles, Illumina, San Diego, CA, USA) was used for sequencing. The libraries (final concentration of 4 nM, pooled by equal molarity) were denatured by adding 0.2 nM NaOH and diluted to 40 pM with a hybridization buffer from Illumina (San Diego, CA, USA). The final loading concentration was 10 pM libraries and 5% PhiX. Sequencing was conducted according to the MiSeq instruction manual. Captured libraries were sequenced in a multiplexed fashion with a paired-end run to obtain 2 × 150 bp reads with at least 250X depth of coverage. The trimmed fastq files were generated using MiSeq reporter (Illumina, San Diego, CA, USA).

Raw sequence data were analyzed with NextGENe software (v.2.4.3.; SoftGenetics, State College, PA, USA) for the presence of single-nucleotide variants (SNVs) as well as insertions and deletions (indels). For the alignment, the human reference genome GRCh37 (equivalent UCSC version hg19) was built. The sequence quality for each sample was assessed and the cutoff was set to 5% variant allele frequency (VAF).

### 2.5. In Silico Protein Analysis

Protein information for p53 protein (P04637, P53_HUMAN) was obtained from UniProt database and RCSB Protein Data Bank. The 3D structures of truncated proteins were built by Robetta protein structure prediction software [12,13]. The p53 protein post-translational modification (PTM) sites affected by mutations were identified using the PhosphoSite Plus web page [14]. Disorder prediction was performed by IUPred3 web server [15]. GORIV was used for secondary structure prediction [16]. Protein sequence-based (Seq) stability changes of variants were predicted by I-Mutant2.0 [17] and DDGun [18,19] web servers, using default parameters. To perform structure-based (struc) stability tests we used I-Mutant2.0 [17], DynaMut2 [20], and DDGun3D [18,19] applications. Predictions were performed by using the following p53 protein crystal structures (Cry): 5MCT, 5MG7, 5MF7 [21], 1AIE [22], 1C26 [23], 2FOO [24], 1YC5 [25], and 3D model built by the highly accurate deep learning algorithm AlphaFold (AF) [26,27]. The predicted effects of the mutations on protein stability (ΔΔG_stability_ value) were compared with experimentally determined changes only in the case of p.G245S [28] and p.R248Q [29] mutations, as such experimental data were available only for these mutants.

To study the protein–protein interactions (PPI), mCSM-PPI2 [30] method was used to calculate the interactions of p53 monomers for each other in a tetrameric structure in the case of p.G334R mutation, and to determine the interaction with ubiquitin carboxyl-terminal hydrolase 7 (or herpesvirus-associated ubiquitin-specific protease) (USP7/HAUSP) protein in the case of p.S362N mutation, as well. The 2FOO [24] crystal structure was used for PPI analysis in the case of p.G334R mutation because this crystal structure contained the N-terminal domain of USP7/HAUSP complexed with p53 peptide. In the case of p.S362N mutation, we used crystal structures containing the oligomerization domain of tetrameric p53 as follows: 1OLG [31] and 1SAL [32]. Changes in the affinities of mutant p53 proteins for DNA were predicted by using mCSM-NA [33].

To determine the aberrant proteins’ pathogenicity, we used the National Cancer Institute (NCI) *TP53* Database (R20, July 2019) [34], its components (Align-GVGD [35,36], BayesDel [37,38], REVEL [39], Sift class [40], PolyPhen2 [41], Transactivation, TransactivationClass [42], DNE_LOFclass [43], DNE class [44,45], Structure/Function class [42]), ClinVar [46,47,48,49,50], Varity [51], Phd-SNP^g^ [52], and FATHMM-XF [53], as well.

### 2.6. Statistical Analysis

Statistical analyses were performed with GraphPad Prism 8.0.1. for Windows (GraphPad Software, San Diego, CA, USA). One-way ANOVA followed by Tukey’s multiple comparisons test was performed to study differences in p53 protein pathogenicity between the three groups (AML-MR, MDS-IB, MDS-LB) in the case of collected mutation data (*TP53* database, Varity, Phd-SNP^g^, FATHMM-XF) and stability analysis. Correlation Matrix construction (correlation coefficient Pearson r) was performed to determine the association among the stability prediction methods (I-Mutant2.0, DynaMut2, DDGun, and DDGun3D). The value of *p* < 0.05 was considered to be statistically significant.

## 3. Results

### 3.1. Patients Clinicopathological Characteristics

The mean age of the patients was 64.1 (range: 25–90). The average age of the three subgroups was 64.7 (AML-MR, range: 33–88), 63.1 (MDS-IB, range: 25–90), and 63.9 (MDS-LB, range: 25–89) years. The female/male ratio was 42/35 (AML-MR: 13/13, MDS-IB: 4/8, MDS-LB: 25/14). The female average age was 68.85 (AML-MR, range: 47–88), 62.50 (MDS-IB, range: 25–90), and 68.88 (MDS-LB, range: 43–89), while the mean male age was 60.62 (AML-MR, range: 33–78), 63.38 (MDS-IB, range: 48–90), and 55.07 (MDS-LB, range: 25–77). The p53 IHC, NGS, cytogenetic results, and OS of the cases are shown in Table 1.

### 3.2. Next-Generation Sequencing

**Figure 1:**
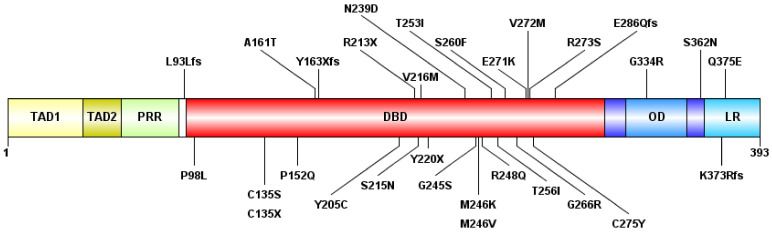
**The schematic representation of the p53 protein structure and the detected mutations.** The following domains are shown: transactivation domain 1 (TAD1) (1–40 amino-acid, AA), TAD2 (40–60 AA), proline-rich region (PRR) (60–90 AA), DNA-binding domain (DBD) (94–312 AA), oligomerization domain (OD) (323–355 AA), and lysin-rich C-terminal tail (LR) (364–393 AA). “fs”: type of AA change is a frameshift, “X”: termination codon.

Out of the 77 cases, we found at least one *TP53* mutation in 26 cases and detected in a total of 41 mutations (Figure 1), including 30 different genotypes. By group, 15 of 26 AML-MR samples (57.69%), four of 12 (33.33%) MDS-IB samples, and seven of 39 (17.95%) MDS-LB samples were found to have *TP53* gene aberrations. In seven cases, two or more mutations were found within a single sample, five in the AML-MR group and one in the MDS-IB, and one in the MDS-LB groups. The total average variant allele frequency (VAF) was 22.75%. The VAF was 34.12% in the AML-MR, 35.59% in the MDS-IB, and 22.83% in the MDS-LB group. The NGS results are summarized in Table 2.

**Figure 2:**
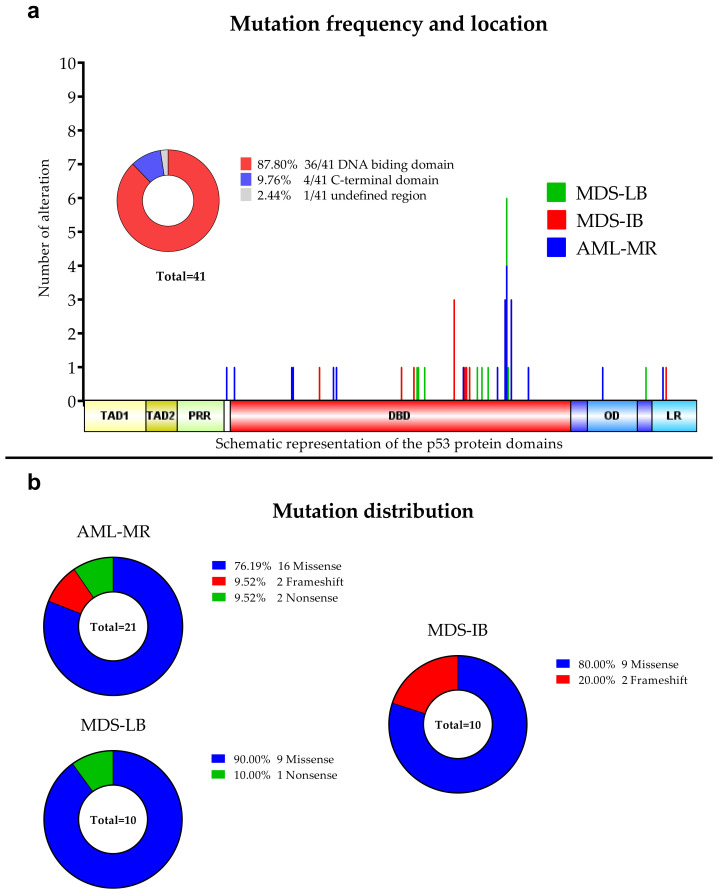
**Detected TP53 aberrations using NGS.** (**a**): Mutation frequencies in the three patients’ group. The locations of the detected alterations are shown for the p53 protein. (**b**): Distribution of the mutations among the three subgroups.

**Figure 3:**
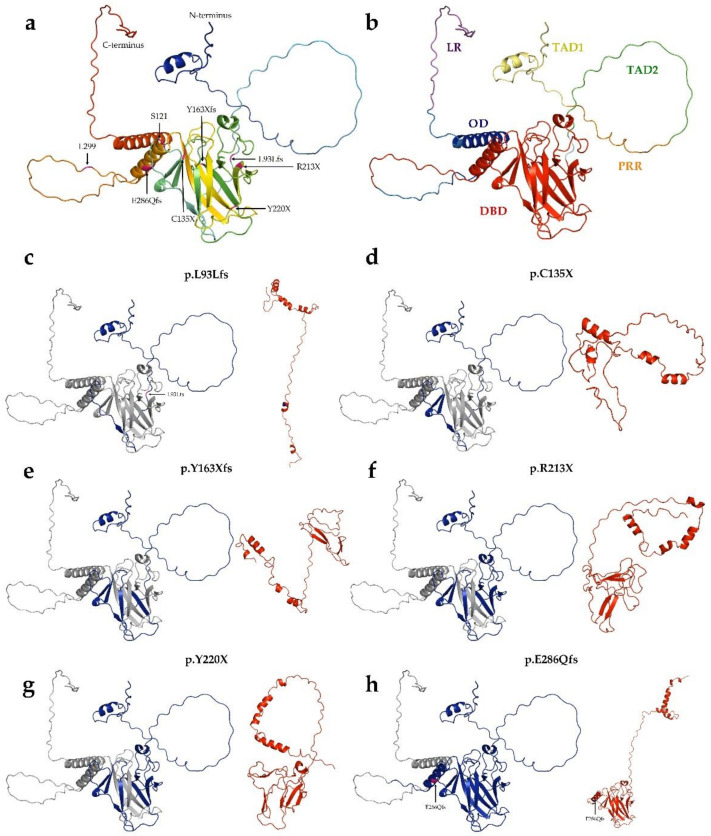
**Structure of p53 Robetta prediction of truncated proteins.** (**a**): Tertiary structure of wild-type p53 protein. The structure was generated by AlphaFold. (**b**): Overall structure and domain organization of wild-type p53. (**c**–**h**): The wild-type protein was colored blue for the length of its aligned mutant-type (red) counterpart. (**c**): p.L93Lfs (121 AA) protein, (**d**): p.C135X (134 AA), (**e**): p.Y163Xfs (162 AA), (**f**): p.R213X (212 AA), (**g**): Y220X (219 AA), (**h**): p.E286Qfs (299 AA).

Of the 30 types of mutations, 25 (83.3%) were located at the DNA binding domain (DBD) (Figure 1 and Figure 2). Of the 30 different *TP53* mutations, 23 (77%) were missense variants, four (13%) were frameshift variants, and three (10%) resulted in a stop codon. Out of the seven non-missense mutations, in six cases the length of the protein product was compromised, resulting in a truncated protein (Figure 3).

### 3.3. Comparison of the Cytogenetic, IHC Results, OS, and TP53 Mutation Status

Out of all *TP53* mutant cases, 11/26 (42.3%) had a complex karyotype (CK). Of the 15 *TP53* mutant AML-MR patients, 9 were CK (60%). In the MDS-IB group, 4/2 (50%) cases were proven CK, while in the MDS-LB mutant positive group, no CK was detected, respectively (cytogenetic analysis was not performed in 12 cases). In the AML-MR group, 15/26 (57.7%) cytogenetic aberrations were detected, while in the MDS groups the chromosome alterations were proven with lower frequencies (4/12—33.3% in MDS-IB and 7/38—18.4% in MDS-LB patients, respectively).

IHC staining was considered to be positive in a total of 13 cases (16.9%), with 10 (38.5%) positive in the AML-MR group, two (16.7%) in MDS-IB, and 1 (2.6%) in MDS-LB. In total, 14.3% of the cases were IHC and NGS positive, while 42% of all NGS mutants were IHC positive. Nine of 15 NGS mutants were IHC positive in the AML-MR group (60%), 50% in the MDS-IB group, and no IHC and NGS double-positive cases were found in the MDS-LB patients. Of the six samples that resulted in truncated proteins, five had negative staining results following IHC. The p.R213X, p.Y163Xfs, p.E286Qfs, p.L93Lfs as single mutations, p.C135X with another two mutations within one sample, were IHC negative, while p.Y220X in parallel with another mutation was IHC positive.

**Figure 4:**
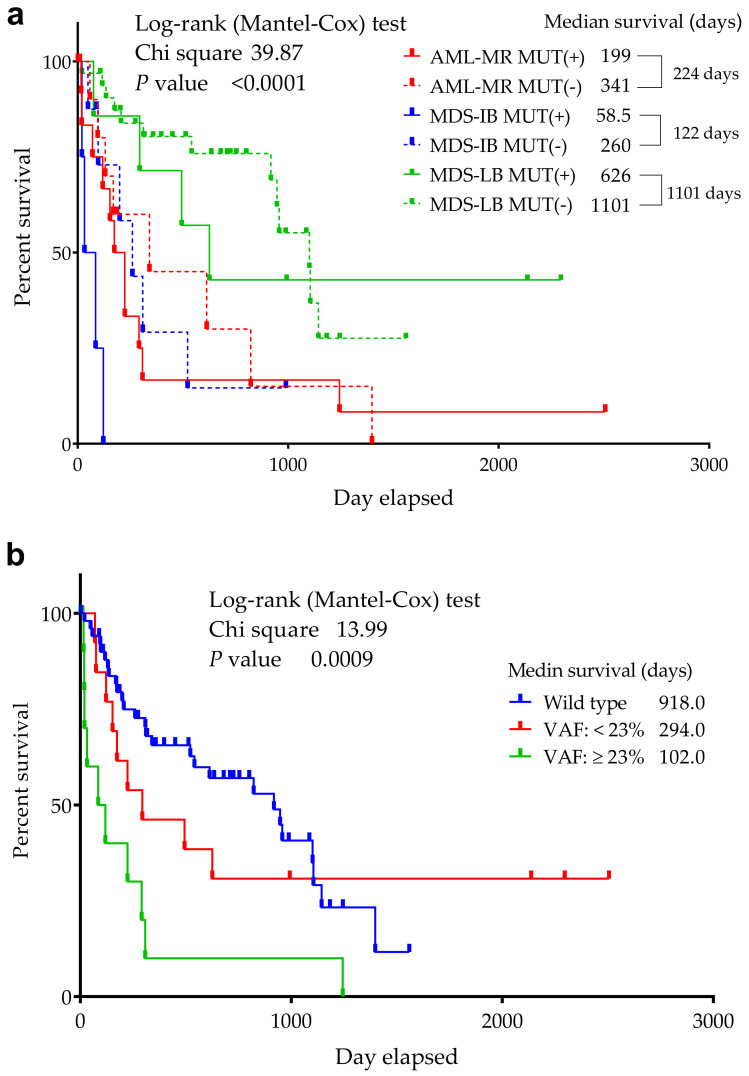
**Overall survival of *TP53* mutant and non-mutant (wild-type) AML/MDS patients.** The abscissa axis shows the survival of the patients in days. The ordinate axis indicates the group’s remaining survivals (%) of all group members. (**a**): Patients are divided into six groups according to mutation status and diagnosis. (**b**): Patients are divided according to VAF distribution. A VAF > 23% had an increased risk of death compared to wild-type patients, while cases with a VAF ≤ 23% had a similar OS to wild-type patients [54].

A significant difference was found in the median OS between the 3 groups (*p* ≤ 0.0001) in the respect of mutant/wild *TP53* status (Figure 4) (918 days for wild-type cases—AML-MR: 341, MDS-IB: 260, MDS-LB: 1101, while 224 days for mutant patients—AML-MR: 199, MDS-IB: 58.5, MDS-LB: 626 days, as well).

### 3.4. Mutations’ Pathogenicity

The six truncated proteins (Figure 3) were considered to be non-functional due to the lack of the C-terminal domain. It was not possible to perform all stability studies or determine the extent of their pathogenicity. Although the length of p.K373Rfs is identical to that of the wild-type protein, the sequence is highly different after the frameshift mutation; therefore, it was not comparable with other missense SNP mutants in subsequent stability and pathogenicity scoring systems.

**Figure 5:**
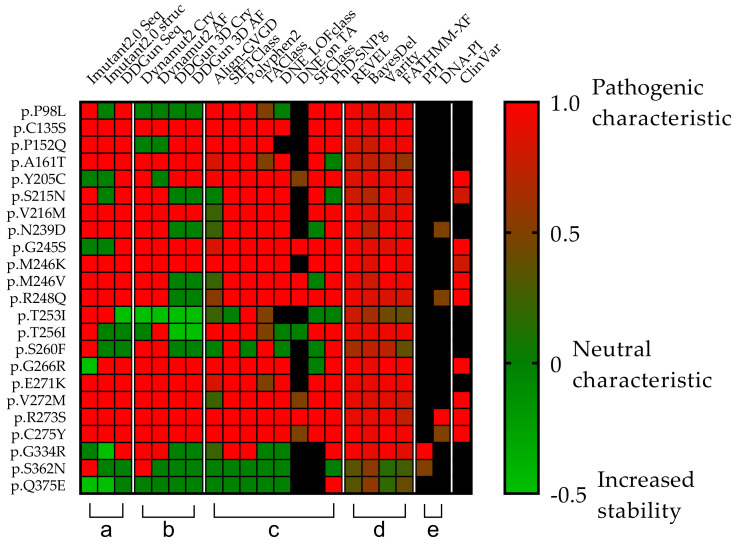
**Comparison of the clinical relevance of the detected p53 mutations.** Each row represents a p53 missense mutation, yet the columns represent protein stability predictions (**a**,**b**), different scoring systems (**c**,**d**), and interaction affinity studies (**e**) universally denoted by a color transition from green to red and corresponding value from 0 to 1, where the two endpoints are green = non-pathogenic and red = pathogenic). (**a**,**b**) Seq means sequence basis methods (**I-Mutant2.0 seq**, **DDGun seq**), Cry means wild-type protein crystal structures (**I-Mutant2.0 Cry**, **DynaMut2 Cry**, **DDGun 3D Cry**), and **AF** means protein structures generated with AlphaFold AI (DynaMut2 AF, DDGun 3D AF). Stability change was considered to be decreasing, if it was less than −0.5 kcal/mol (score 1, red) and increasing (score −0.5, light green) if it was higher than 0.5 kcal/mol, otherwise, the stability change of the mutation was considered as neutral (score 0, green). (**c**,**d**) **Align-GVG** represents the original C0 to C65 order on a color scale, where 0 = C0 and 1 = C65. **SIFTCLass** uses the pathogenicity class defined by Sift class, in the table 0 = benign, 1 = damaging. **Polyphen2** class Benign = 0, possibly damaging = 0.5, probably damaging = 1. **TAClass** uses TransactivationClass classification, which divides variants into three functional categories “functional”, which is 0 in the figure, “partially functional” with a value of 0.5, and “non-functional” with a value of 1. **DNE LOFclass** indicates whether the mutation has a dominant-negative effect and loss-of-function effect (if yes, the value is 1, if no, the value is 0). **DNE on TA** uses scoring categories, whether the mutant has a dominant-negative (DN) effect on the transactivation of wild-type p53 (if the answer is “yes” the value is 1 if “moderate” the value is 0.5). **SFClass** (Structure Function Class) classifies the structural functionality of the mutant into non-functional (score 0) and functional (score 1). **Phd_SNP^g^** predicts human deleterious SNPs in the human genome and is a binary classifier for predicting pathogenic variants in the coding and non-coding regions (0 = non-pathogenic and 1 = pathogenic). **REVEL** scores for an individual missense variant range from 0 to 1, with higher scores reflecting a greater likelihood that the variant is disease-causing. **BayesDel’s** original numbering scheme ranging from −1.29334 to 0.75731 is represented as 0–1, where 0 = −1.29334 and 0.75731 = 1. **Varity** and **FATHMM-XF** score is a predictor of variants pathogenicity scoring from 0 to 1, as well. (**e**) **PPI** shows the protein–protein interactions affinity changes with the value of 0.5 if the rate of decrease was below 0.5 kcal/mol and 1 if it was higher. **DNA-PI** shows the affinity change of the interaction between DNA and p53 protein. The score is 1 (red) when the decrease is less than −0.5-0.5 if not. **ClinVar** column filled in based on the ClinVar database. ClinVar is displayed for comparison and to show that many of the *TP53* mutations in the database are missing or not classified. Only those variants were assigned a value as pathogenic (score 1) or likely pathogenic (0.8), even “Conflicting interpretations of pathogenicity” mutants with “Uncertain significance” and variants not found in the database were not assigned a value Our table was based on data from the TP53 Database supplemented with scores from Varity and FATHMM-XF. Black squares were shown as missing information in the databases.

The clinical relevance of the 23 different mutations was first Investigated in the ClinVar database, which identified seven as pathogenic, two as likely pathogenic, one as pathogenic/likely pathogenic, while 11 were undetermined (six “Uncertain significance”, five “Conflicting interpretations of pathogenicity”). Two mutations were not included in the database (p.S260F and p.Q375E). Due to the uncertainties of the data, we examined the 23 mutations in downstream analyses by using different databases and scoring systems (Figure 5).

### 3.5. In Vitro Experiments in the TP53 Mutation Database

The IARC TP53 database contains eight types (WAF1, MDM2, BAX, h1433s, AIP1, GADD45, NOXA, P53R2) of promoter-specific transcriptional activity measured in yeast functional assays and expressed as a percent of wild-type activity. Out of 30 types of mutations we found, no data are available for p.L93fs, p.Y163fs, p.E286Qfs, and p.K373fs. The 26 accessible mutations can be split into two groups by domain (23 in DBD and three in the C-terminal of the protein). On average, mutations found in DBD have only 13.84% of the promoter-specific transcriptional activity compared to the wild-type protein (range: 0.09% in the case of p.C275Y and 40.24% in the case of p.P98L), yet the p.G334R, p.S362N, and p.Q375E variants detected in the C-terminal domain have an activity of 87.72%, 78.06%, and 80.33%, respectively.

In vitro experiments reveal that p.C135X is loss-of-function in growth suppression as well as p.C135S, p.A161T, p.Y163fs, p.Y205C, p.R213X, p.S215N, p.V216M, p.Y220X, p.N239D, p.G245S, p.M246K, p.M246V, and p.R248Q variants. One mutation, p.C135S, is LOF in the induction of apoptosis and gain-of-function (GOF) in cooperation with *RAS* or another transformant oncogene, in addition to p.Y205C, p.G245S, p.M246V, p.R248Q, and p.C275Y variants. In the case of a dominant negative effect, seven mutations (p.G245S, p.M246V, p.R248Q, p.G266R, p.V272M, p.R273S, p.C275Y) have this kind of pathogenicity. According to the database, mutations p.G245S, p.R248Q, p.G266R, and p.R273S are GOF and consequently counteract p73 activity when both proteins are expressed in a cell system.

### 3.6. Mutant p53 Protein Stability Analysis

**Figure 6:**
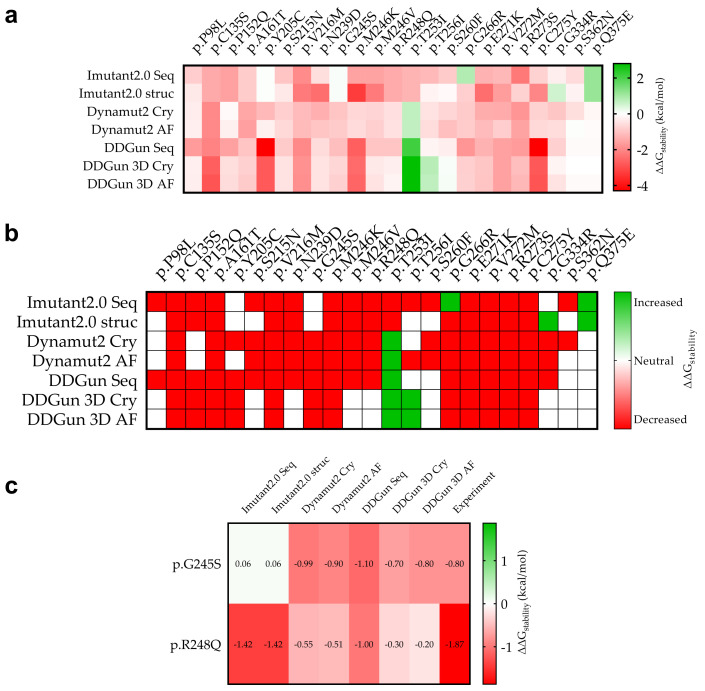
**Stability change (**ΔΔG_stability_**) due to mutation of p53 mutant proteins and experimental comparison**. (**a**): The specific calculated results are shown in red (decreasing stability) and green (increasing stability). The figure shows for which mutations we calculated decreasing and increasing ΔΔGstability in mutant proteins in kcal/mol as a result of the mutations. (**b**): The stability change was considered as decreasing if it was less than −0.5 kcal/mol (red) and increasing (green) if it was greater than 0.5 kcal/mol, otherwise the stability change of the mutation was considered to be neutral (white). (**c**): Two mutations p.G245S and p.R248Q that have been measured experimentally so far and their predictive comparison (see Section 2).

In silico analyses were performed to determine the effect of sequence variants on the stability of the p53 protein (Figure 6) using I-Mutant2.0, DynaMut2, and DDGun analysis tools. The study was performed based on the sequence (I-Mutant2.0 seq, DDGun seq) or structure of the protein, by using crystal structures of the wild-type protein (I-Mutant2.0 Cry, DynaMut2 Cry, DDGun 3D Cry) and a model structure generated by AlphaFold AI (DynaMut2 AF, DDGun 3D AF).

A comparison was performed between our predicted and the available experimental results (Figure 6c). For p.G245S, we obtained an exact match (DDGun 3D AF), while in the case of p.R248Q, similar to the experimental result, the stability change was decreased.

**Figure 7:**
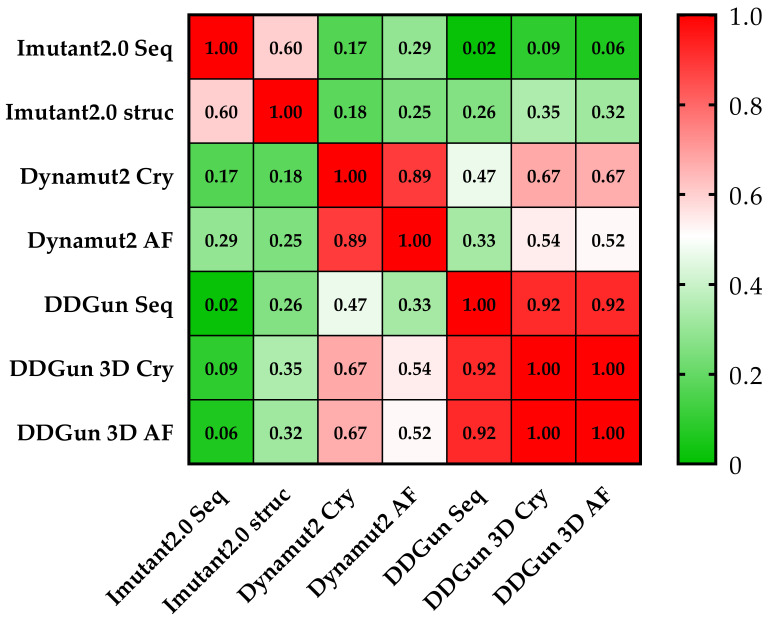
**Pearson correlation matrix of the seven prediction methods.** None of the results obtained by the I-Mutant 2.0 analyses showed a correlation of (<0.5 Pearson’s r was observed) with the results of other methods. The cells of the table are colored based on Pearson’s r values (see color scale). The other sequence-based method DDGun seq even with its DDGun pairs is highly correlated with DynaMut2 methods not reaching 0.5.

Similar results were proven using the same methods (DynaMut2 and DDGun 3D) for the analysis of crystal structure (Cry) and model structure (AF), but significant differences were observed between the obtained predictions plotted on a correlation matrix between the different methods (Figure 7). Therefore, we filtered out those methods that had correlations below 0.5 Pearson r with the other members of the matrix (I-Mutant 2.0 seq and struc, DDGun seq). Based on the results of the four remaining predictions (performed by DynaMut2, DDGun 3D tools), we detected significant differences between the changes in stability (ΔΔG_stability_) of p53 proteins in the three clinical groups (AML-MR, MDS-IB, and MDS-LB) in response to mutations using one-way ANOVA statistics.

### 3.7. Changes in Protein-Protein Interactions upon Mutations

**Figure 8:**
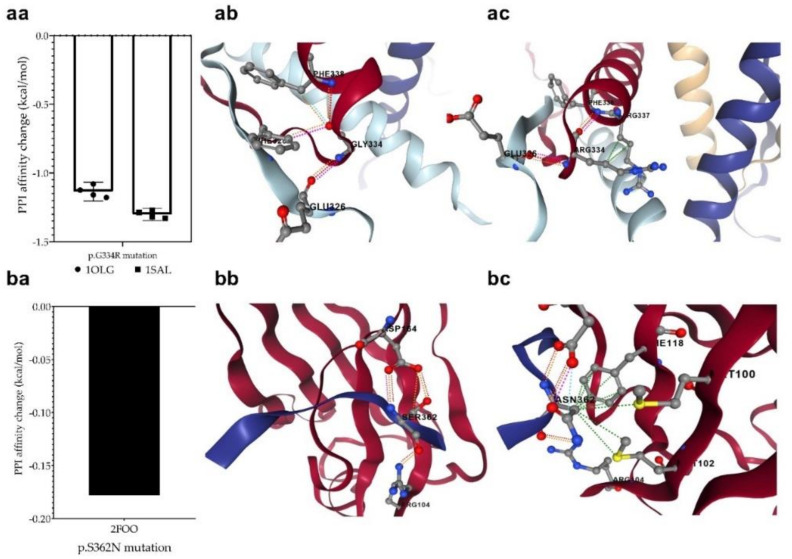
**Changes in protein**-**protein interactions (PPI) upon p.G334R (aa) and p.S362N (ba) mutations**. The 1OLG, 1SAL, and 2FOO are the PDB identifiers of the studied crystal structures (see Section 2). P.G334R mutation was supposed to affect interactions between the monomers of tetrameric p53, while p.S362N was considered to affect the interaction of p53 and USP7/HAUSP proteins. (**ab**): PPI between wild-type chains, (**ac**): PPI between wild-type and mutant chain, (**bb**): PPI between wild-type protein chain and USP7, (**bc**): PPI between mutant protein and USP7. The different dash dots between the residues indicate the type of interactions. Pink: clashes; light blue: van der Waals; red: hydrogen bond; yellow: ionic bond; green: hydrophobic; orange: polar.

The p.G334R mutation (detected in the MDS-LB group) is located in the oligomerization domain and therefore is supposed to affect the tetramerization of p53 monomers. The mCSM-PPI2 program was used to investigate the effect of the mutation on the interaction between the monomers in the formation of the tetrameric structure. For this purpose, we used two p53 protein crystal structures having a tetrameric conformation (1OLG and 1SAL). We predicted a decreased affinity (−1.218 kcal/mol on average), based on the analysis of four-four chains of two crystal structures. This mutation may potentially weaken the interactions between the monomers in the process of oligomerization (Figure 8a).

We also detected another mutation at a position that may contribute to protein–protein interactions. The p.S362N mutation in the MDS-LB group is located at the interaction site of p53 and USP7/HAUSP (359–362) proteins. To find out whether the mutations affect this interaction, we predicted the change in the interaction energy (−0.178 kcal/mol (Figure 8b) using the mCSM-PPI2 program. The interaction of the two proteins is likely to be affected by the mutation at this interaction site, resulting in a different interaction strength than normal.

### 3.8. Changes in p53 Protein-DNA Interactions as Affected by Mutations

**Figure 9:**
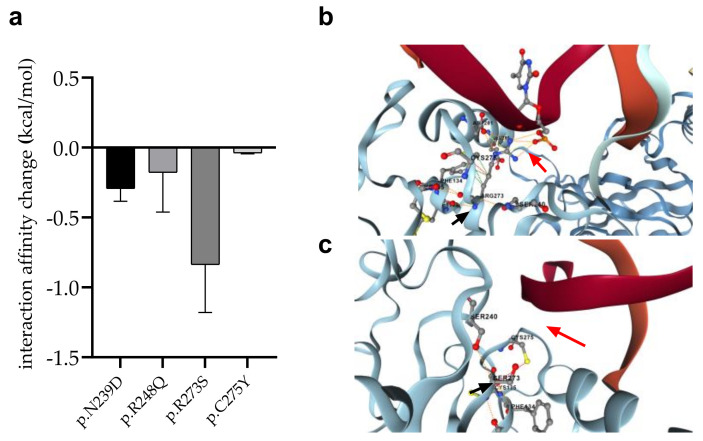
**Changes of interaction between p53 and DNA in response to mutations.** (**a**): Error bars indicate SD of the values obtained from the analysis of three different structures. (**b**): DNA-protein interaction (red arrow) between p.R273 (black arrow) wild-type protein and DNA, (**c**): Absence of DNA-protein interaction (red arrow) between p.R273S mutant (black arrow) protein and DNA.

We detected four mutations (p.R273S in AML-MR; p.C275Y and p.N239D in AML-MR and MDS-LB; p.R248Q in MDS-IB patients, respectively) of such residues, which directly contribute to the interaction of p53 with DNA. Using the mCSM-NA program, three different crystal structures of p53-DNA complexes (see Section 2) were examined to predict changes in intermonomeric interactions (Figure 9).

In almost all cases, the mutations were predicted to weaken the interaction between p53 and DNA. The most remarkable decrease was observed for the p.R273S (−0.8370 kcal/mol), while only a moderate change was calculated for the p.C275Y mutation (−0.03867 kcal/mol).

### 3.9. Statistical Analyses of the Mutant p53 Protein Pathogenicity and Structural Stability in the AML/MDS Patients

**Figure 10:**
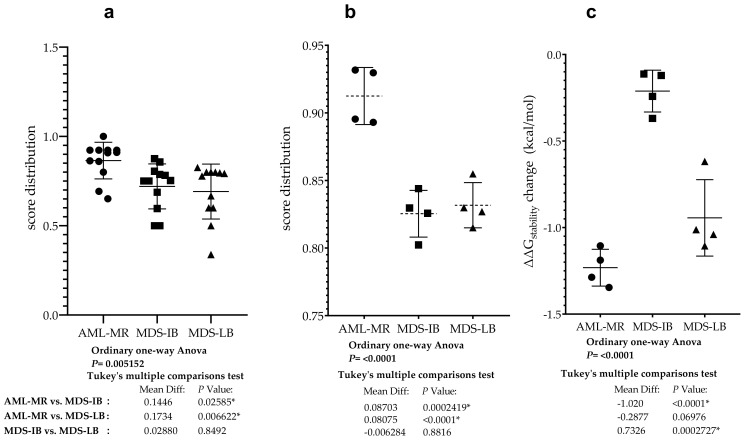
Differences between AML/MDS patients in the sight of mutant p53 protein pathogenicity and structural stability. (**a**): Comparison of the severity of p53 mutations found in AML-MR, MDS-IB, and MDS-LB cases. Significant differences were found between two of the three members based and scores in Figure 5c,d. (**b**): Comparison of p53 mutations’ pathogenicity in the three groups using REVEl, BayesDel, Varity, and FATHMM-XF scores (Figure 5d). (**c**): Statistical differences between the three patients’ groups according to the changes in p53 protein stability predicted by their mutations with methods from Figure 5b. * indicate the statistically significant differences, *p* values are described in the text.

To compare the pathogenic status of the three different groups using specific scores based on databases see Figure 5c,d, a one-way ANOVA was performed (Figure 10a). There was a statistically significant difference in mean score between at least two groups (F(2, 33) = [6.207], *p* = 0.0052). Tukey’s multiple comparison test found that the mean score was significantly different between AML-MR vs. MDS-LB (*p* = 0.0066, 95% C.I. = [0.04396–0.3027]) and AML-MR vs. MDS-IB (*p* = 0.0259, 95% C.I. = [0.01516–0.2739]). There was no statistically significant difference in mean scores between MDS-IB vs. MDS-LB (0.8492).

Analysis was performed specifically using four methods where all data for mutations were available and pathogenicity classifications were originally non-categorical (Figure 5c). Using scores from three different clinical groups, REVEL, BayesDel, Varity, and FATHMM-XF, a one-way ANOVA was performed to compare pathogen status (Figure 10b). There was a statistically significant difference in mean score between at least two groups (F(2, 9) = [27.66], *p* = 0.0001). The Tukey’s multiple comparisons test found that the mean score was significantly different between AML-MR vs. MDS-IB (*p* = 0.0002, 95% C.I. = [0.05059–0.1235]) and AML-MR vs. MDS-LB (*p* = 0.0004, 95% C.I. = [0.04431–0.1172]). There was no statistically significant difference in mean scores between MDS-IB vs. MDS-LB (*p* = 0.8816).

Four prediction methods were used to analyze the differences in structural stabilities between the three AML/MDS groups. To perform this analysis, we used DynaMut2 Cry (crystal structure-based) and AF (AlphaFold), DDGun 3D Cry, and AF. The I-Mutant2.0 Seq, I-Mutant2.0 struc, and DDGun Seq were not included in the analyses because of the low Pearson r value in the correlation matrix (Figure 7). A one-way ANOVA (Figure 10c) revealed that there was a statistically significant difference in mean score between at least two groups (F(2, 9) = [44.44], *p* ≤ 0.0001). The Tukey’s multiple comparisons test found that the mean value of the score was significantly different between AML-MR vs. MDS-IB (*p* < 0.0001, 95% C.I. = [−1.332–−0.7087]) and MDS-IB vs. MDS-LB (*p* 0.0003, 95% C.I. = [0.4210–1.044]). There was no statistically significant difference between AML-MR vs. MDS-LB (*p* = 0.0698).

## 4. Discussion

In the area of precision oncology, high-throughput molecular analysis has become essential to identify biologically distinct disease subgroups and tailor the most effective treatment options. MDS is a group of clonal hematopoietic stem cell disorders frequently progressing to AML, as such considered a pre-leukemic condition. Somatic *TP53* gene mutations are key determinants of progression and disease survival in MDS/AML patients [1]. MDS patients with *TP53* mutations represent a distinct molecular cohort with uniformly poor prognosis, however, the *TP53* mutation status remained the most important additional risk factor not considered by the currently existing prognostic scoring systems [9,10,11,55,56]. The mechanisms by which *TP53* mutations drive these inferior outcomes have not been resolved.

In the present study, ultra-deep NGS analysis targeting the *TP53* gene was performed on all samples of 77 AML-MR, MDS-IB, and MDS-LB patients. In total, 26 patients with *TP53* mutations were found, and 30 differential variations were identified in a total of 41 mutations. The highest proportion of *TP53* mutations was detected in AML cases (57.69%), followed by 33.33% in MDS-IB samples and 17.95% in MDS-LB samples, all having *TP53* gene aberrations. In the comparison of the *TP53* mutation status and cytogenetic landscape, following the literature [57,58], we detected the most cytogenetic aberrations and *TP53* mutations in the AML and MDS-IB groups. In the mutant AML-MR patients, 60% were associated with CK, in the MDS-IB group, 50%, while in the MDS-LB mutant positive patients, no cases of CK were detected.

By considering VAF and the number of alterations together, we could predict whether single or multiple clones were present. In seven cases, two or more mutations were found within the same sample. Similar VAF (case 1, 10, 13, 32, 42) suggests the possibility of parallel mutations in both of the *TP53* alleles (compound heterozygote) in a single clone or more mutations in the same allele considered as multi-hit status [58]. Two or more *TP53* mutations detected with different abundance suggest that they are derived from different clones (cases 6 and 28).

The prognostic significance of *TP53* mutations depends in part on their variant allele frequency (VAF), with less frequent clones having a less adverse impact [59,60]. Patients with a lower VAF had better survival, according to the literature, *TP53* mutant cases with a VAF > 23% had an increased risk of death compared to wild-type patients, while cases with a VAF ≤ 23% had a similar OS to wild-type patients [54]. Our results revealed a higher average VAF in AML-MR and MDS-IB groups as compared to MDS-LB cases (34.12% and 35.59% vs. 22.83%, respectively).

Mutant type p53 immunopositivity was defined following IHC when p53 staining intensity was high (3+) with at least 10% of positive cell positivity stated in a total of 13/77 cases (16.9%). Patients without *TP53* mutations did not have a strong IHC for p53, conferring a good negative predictive value for IHC. In the presence of cases with only the protein-truncating mutation (cases 5, 16, 29, and 60), IHC did not detect the altered p53 protein because the damage reduces the protein half-life.

The in silico structural analysis of mutant p53 proteins may reveal the association of p53 with the progression of AML/MDS at the protein level. The alteration of the structure by point mutations potentially affects protein function, and the predicted structural changes of the p53 protein may correlate with the clinical behavior in a clonal fashion. Different classes of mutations are expected to cause distinct effects, which can be predicted by sequence—as well as structure-based computational approaches. For example, not all the mutations in the DNA-binding domain are necessarily loss-of-function mutations. These categories are predicated on the location of the mutation within the N-terminal, DNA-binding, or oligomerization domain, as well as the often context-dependent effects of the mutation on p53 function as follows: complete or partial loss of function, a dominant-negative effect, and/or gain-of-function properties [61]. In total, 87.8% of the *TP53* mutations (36) were detected in the DBD region of the protein.

In silico bioinformatic methods were approached to validate the most frequent hot spot *TP53* mutations in the applied databases; however, the effect of rare mutations on AML/MDS is still largely unknown. For this reason, we also examined the pathogenicity of the *TP53* mutation to acquire new information in terms of database data, and by performing an analysis of stability and interactions using established biostatistical algorithms. In total, 20 out of the detected 30 types of mutations are currently not categorized in the ClinVar database; thus, their clinical significance remains mysterious. Therefore, in silico analysis and data collection were performed to predict the variant’s pathogenicity. We found significant differences between AML-MR vs. MDS-IB and AML-MR vs. MDS-LB groups based on 12 scoring methods, and the same significant differences in scoring on REVEL BayesDel, Varity, and FATHMM-XF pathogenicity scores. Stability assays (DynaMut2, DDGun) also revealed differences in ΔΔG_stability_ (kcal/mol) due to mutations, although significant differences (among the three patient groups) were detected only between the AML-MR vs. MDS-IB and MDS-IB vs. MDS-LB groups. The lack of a significant difference between the clinically more severe AML-MR and milder MDS-LB, and the significantly lower mean stability of mutations in the MDS-LB group, may be the result of the low sample size of the MDS-IB group. At the same time, both the pathogenicity scoring and stability change prediction methods were able to distinguish, to varying degrees, between the apparent pathogenicity of the mutations in the three groups.

We found that out of the 30 types of mutations, 15 variants (p.E271K, p.S260F, p.T256I, p.P98L, p.Q375E, p.T253I, p.R248Q, p.P152Q, p.S362N, p.N239D, p.C275Y, p.V272M, p.M246V, p.M246K,p.R273S, p.G266R, p.A161T, p.C135S, p.V216M, p.G245S, p.S215N, p.Y205C, and p.G334R) predicted decreased stability values, seven (p.S260F, p.T256I, p.P98L, p.Q375E, p.T253I, p.R248Q, p.P152Q, and p.S362N) variants were neutral, and one variant (p.T253I) showed an increase in aberrant p53 protein stability compared to the normal genotype. Some p53 mutant proteins with decreased stability were investigated in clinical studies [62,63], where the worse outcome was proven with complex chromosome aberration. Out of the remaining seven non-missense mutations, six (four frameshifts, two stop codon mutations) variants result in truncated proteins that have lost the entire C-terminal domain and have truncated DBDs. These proteins are most likely non-functional and may have degraded immediately after translation. Divided into groups, we observed that in the AML-MR group with a worse prognosis, 12 of the 13 missense mutations predicted to exhibit decreased p53 protein stability change, two frameshifts, and two stop codon mutations were detected. In this group, all of the seven non-missense mutations and three variants were found at direct protein–DNA interaction sites, which might have resulted in weaker interactions with DNA. In the MDS-IB group, the effect of six of the eight missense variants was predicted to be neutral on the protein; two decreased and one increased its stability. Two frameshift mutations and one protein–DNA interaction partner were also included in this group. In the MDS-LB group with the best prognosis, four of the five missense variants were predicted to have decreased stability and one was classified as neutral, in addition to one-stop codon mutation. Interestingly, both the two mutations tested in the PPI study (p.G334R and p.S362N) were observed in this group.

In silico analyses were used to calculate the possible changes in protein–protein and protein–DNA interactions. These analyses revealed that the p.G334R mutation of the oligomerization domain may reduce the intermonomeric interaction between the p53 monomers (−1.218 kcal/mL), which may have an impact on tetrameric structure formation. Furthermore, the p.S362N mutation, already addressed by other studies [24], prevents the interaction between USP7/HAUSP and the p53 protein. Ser362 not only interacts with USP7/HAUSP but also functions as a PTM site (phosphorylation). Replacement at Ser-362 and Ser-366 with alanine results in a decrease in phosphorylation of p53 by IKK2 and a decrease in association with TrCP1, and thus an increase in p53 stability and p53 target gene, altering the G1 phase of the cell cycle [64]. Another mutation we have found that serves as a PTM site is p.Ser215 (p.S215N). The p.Ser215 is a PAK4 kinase phosphorylation site; modification at this site leads to a decrease in p53 activity in hepatocellular carcinoma cells [65]. The impact of the loss of this modification in MDS-IB cases is not yet known.

The promoter-specific transcriptional activity [42,66,67] reflects our in silico results showing that the p.S362N and p.Q375E variants had the lowest pathogenicity characteristics. The p.G334R variant had the most wild-type promoter-specific transcriptional activity, and in vitro data suggests that it is capable of forming a tetrameric structure despite the decrease in silico PPI affinity predicted by our data.

In the case of the p.T253I mutation, the methods we used (Figure 5b) predict increased p53 protein stability, in contrast to the mixed results of the pathogenicity classification systems (Figure 6c,d), indicating a partially functional mutant protein (TransactivationClass [42]). The p.S362N is a benign variant according to almost all the methods shown in Figure 5, so then, as described above, the p.S362 is a PTM site; therefore, the p.S362N mutation may prevent the phosphorylation at this site and may potentially affect oligomerization of the p53 protein. Consequently, p.S362N mutation cannot be classified with absolute certainty to be benign. In contrast, p.Q375E is more likely to be a benign mutation based on the data we have collected and the calculations we have performed. The other mutations show pathogenic characteristics even if their protein stability is not decreased according to our predicting methods.

## 5. Conclusions

In the present study, an investigation of the clonal heterogeneity and severity of hematopoietic disorders in MDS and AML samples compared to the *TP53* gene mutation status was performed using in silico approaches. Because of the interpretation difficulties and the absence of clinical data on detected aberrations, pathogenicity was established based on different scoring systems. The largest number of abnormalities with the most severe consequences were found in AML-MR cases. Based on our molecular and protein in silico data, the MDS-IB is an intermediate group between AML-MR and MDS-LB patients, which frequently progresses to AML, and such is considered a pre-leukemic condition. Individual variants with unclear clinical significance can be further evaluated by in silico modeling, enabling the prediction of their pathogen character.

## Figures and Tables

**Table 1:** Clinicopathological results and overall survival (OS) of the patients.

Cases	Sex	Age (Years)	Diagnosis	OS (Days)	p53 IHC *	*TP53* NGS	Karyotype
1	F	65	AML-MR	0	+	+	CK
2	M	73		119	+	+	n.a.
3	M	35		169	-	-	CK
4	F	47		2506	-	+	normal
5	M	76		1244	-	+	CK
6	M	64		308	+	+	n.a.
7	M	69		171	-	-	CK
8	F	76		3	-	-	n.a.
9	M	33		131	+	-	normal
10	M	78		153	-	+	CK
11	F	53		291	+	+	CK
12	M	64		822	-	-	normal
13	F	73		70	-	+	tetrasomy
14	F	88		95	-	-	normal
15	F	83		1	+	+	CK
16	M	66		174	-	+	CK
17	M	61		224	-	+	normal
18	F	64		186	-	-	47,XX,+8
19	F	66		341	-	-	CK
20	M	63		19	+	+	CK
21	F	86		224	+	+	n.a.
22	M	41		58	-	-	normal
23	F	56		613	-	-	CK
24	M	65		10	+	+	CK
25	F	69		16	+	+	CK
26	F	69		1398	-	-	normal
27	M	48	MDS-IB	89	-	-	normal
28	M	56		32	-	+	n.a.
29	M	59		85	-	+	CK
30	F	69		989	-	-	normal
31	F	25		260	-	-	46,XX,t(8;21)/46,XX
32	M	80		20	+	+	CK
33	M	61		199	-	-	normal
34	M	79		122	+	+	n.a.
35	M	59		49	-	-	CK
36	F	90		97	-	-	normal
37	M	65		309	-	-	normal
38	F	66		522	-	-	normal
39	F	52	MDS-LB	626	-	+	normal
40	F	80		312	-	-	normal
41	F	71		494	-	+	46,XX,t(2;12)/46,XX,t(17;17)
42	M	64		2296	-	+	normal
43	F	64		754	-	-	n.a.
44	M	70		450	-	-	normal
45	M	64		918	-	-	normal
46	M	41		294	-	+	n.a.
47	F	74		710	-	-	46,XX,del(20q)
48	F	61		75	-	+	normal
49	F	58		1086	-	-	normal
50	F	60		993	-	+	normal
51	M	36		274	-	-	normal
52	M	60		175	-	-	normal
53	M	72		513	-	-	normal
54	F	71		1105	-	-	normal
55	F	78		1559	-	-	46,XX,del(5q)/46,XX
56	F	64		20	-	-	n.a.
57	F	89		541	-	-	n.a.
58	M	68		1101	-	-	CK
59	M	36		203	-	-	n.a.
60	F	64		2137	-	+	normal
61	M	77		1144	-	-	CK
62	F	43		726	-	-	normal
63	F	74		947	-	-	n.a.
64	M	70		680	-	-	normal
65	M	30		135	-	-	normal
66	M	25		635	-	-	normal
67	F	84		801	-	-	normal
68	F	76		1245	-	-	normal
69	F	75		1183	-	-	normal
70	F	69		988	-	-	normal
71	F	73		957	-	-	normal
72	M	58		397	-	-	normal
73	F	85		108	-	-	46,XX,del(7q)
74	F	46		358	-	-	46,XX,del(5q)/46,XX
75	F	71		206	+	-	normal
76	F	67		317	-	-	normal
77	F	73		117	-	-	normal

AML-MR: acute myeloid leukemia with myelodysplasia-related changes, MDS-IB: myelodysplastic neoplasias with increased-blast, MDS-LB: myelodysplastic neoplasias with low-blast, CK: complex karyotype. * The p53 IHC positivity was defined when p53 staining intensity was high (3+) with at least 10% of positive cells.

**Table 2:** Detected *TP53* mutations and variant allele frequency (VAF).

Cases	Diagnosis	*TP53* Nucleotide Change	p53 AA Change	VAF (%)
**1**	AML-MR	c.811G > A	p.E271K	27.8
		c.715A > G	p.N239D	29.14
**2**		c.824G > A	p.C275Y	88.04
**4**		c.779C > T	p.S260F	16.2
**5**		c.279delG	p.L93Lfs	36.66
**6**		c.814G > A	p.V272M	30.7
		c.660T > G	p.Y220X	8.38
**10**		c.811G > A	p.E271K	6.08
		c.715A > G	p.N239D	6.67
**11**		c.736A > G	p.M246V	28.39
**13**		c.811G > A	p.E271K	8.28
		c.737T > A	p.M246K	8.35
		c.405C > A	p.C135X	5.65
**15**		c.817C > A	p.R273S	41.02
**16**		c.856_869del14	p.E286Qfs	16.96
**17**		c.824G > A	p.C275Y	12
**20**		c.796G > A	p.G266R	41.73
**21**		c.481G > A	p.A161T	73.42
**24**		c.403T > A	p.C135S	45.06
**25**		c.646G > A	p.V216M	39.44
		c.733G > A	p.G245S	38.22
**28**	MDS-IB	c.644G > A	p.S215N	30.65
		c.1118delA	p.K373Rfs	11.59
		c.767C > T	p.T256I	6.82
		c.293C > T	p.P98L	6.25
		c.1123C > G	p.Q375E	5.6
		c.758C > T	p.T253I	5.45
**29**		c.489delC	p.Y163Xfs	70.27
**32**		c.743G > A	p.R248Q	39.93
		c.455C > A	p.P152Q	38.21
**34**		c.614A > G	p.Y205C	13.49
**39**	MDS-LB	c.824G > A	p.C275Y	12.2
**41**		c.814G > A	p.V272M	13.06
**42**		c.1085G > A	p.S362N	5.19
		c.1000G > A	p.G334R	5.04
		c.814G > A	p.V272M	6.27
		c.715A > G	p.N239D	10.81
**46**		c.814G > A	p.V272M	10.42
**48**		c.814G > A	p.V272M	7.52
**50**		c.814G > A	p.V272M	19.77
**60**		c.637C > T	p.R213X	5.94

## Data Availability

The data presented in this study are available on request from the corresponding author. The data are not publicly available to protect the rights of patients.
